# Spontaneous Electrical Activity and Spikes in the Tail of Marine Cercariae

**DOI:** 10.5402/2013/123108

**Published:** 2013-08-19

**Authors:** O. O. Tolstenkov, M. I. Zhukovskaya, V. V. Prokofiev, M. K. S. Gustafsson

**Affiliations:** ^1^A. N. Severtsov Institute of Ecology and Evolution, Centre of Parasitology, Russian Academy of Sciences, Leninsky Prospect 33, Moscow 119071, Russia; ^2^Sechenov Institute of Evolutionary Physiology and Biochemistry, Laboratory of Evolution of Sense Organs, Russian Academy of Sciences, 44 Thorez Avenue, Saint Petersburg 194223, Russia; ^3^Pskov State University, Faculty of Biology, Lenin Square, 2 Pskov 180760, Russia; ^4^Department of Biosciences, Åbo Akademi University, Artillerigatan 6, 20520 Åbo, Finland

## Abstract

Spontaneous electrical activity is recorded in two species of marine cercariae, *Cryptocotyle lingua* and *Himasthla elongata*, with different types of swimming—by glass microelectrode recordings. Slow local field potentials (sLFPs) of low amplitude and fast high amplitude action potentials (APs) are found. The shape of the sLFPs is different in the species and correlates with the type of swimming. Fast high amplitude APs are recorded for the first time in cercariae. The limited number of APs included in the swimming pattern of larva suggests a key role for the spiking neurons in initiating the motility pattern in the cercaria and needs further research.

## 1. Introduction

Free-living trematode larvae, cercariae, are nonfeeding minute organisms whose only role is to find and infect the next host, a step which is crucial for the survival of the parasite species. In the case of important diseases, such as schistosomiasis and cercarial dermatitis, cercariae represent the infective stage penetrating actively the human skin. Much basic information about the nervous system of trematodes is needed before the development of antiparasitic drugs is possible. The finding of the neuromechanisms of initiation and regulation of motility patterns in the larva is among the essential questions to be answered.

Cercariae often demonstrate fast sensory-motor reactions to different stimuli; for reference see Haas [1]. Small size, short life span of the larva organisms, and the absence of visually identified neurons bring technical difficulties in the study of the cercarial nervous system. The basic physiology of larval motility has not been studied in detail. No up-to-date information on the rapid signal transmission by means of action potentials in the nervous system of cercariae is available. Spontaneous electrical activity has been investigated only in cercariae of *Proterometra macrostoma *(Faust, 1918) Horsfall, 1933, by suction electrode recording; for reference see Rowley et al. [2].

Swimming cercariae show distinctive behavioural patterns related to their strategy of host finding and infection and can be divided into two groups: cercariae with a continuous swimming pattern and cercariae with an intermittent type of swimming pattern, where active phases alternate with passive phases when the cercariae soar in the water [1]. The difference in the swimming patterns correlates with the details of morphology in the tail—the larval locomotory organ, which is shown for two species chosen for the present research [3].

Here, we compare the electrical activity in cercariae of *Cryptocotyle lingua, *Creplin 1825, which have an intermittent swimming pattern, with that in the continuously swimming cercariae of *Himasthla elongata *Mehlis, 1831. 

## 2. Materials and Methods

Samples of snails *Littorina littorea, *Linnaeus, 1758, were collected in the littoral zone of Chupa Bay, White Sea at the Marine Biological Station of the Zoological Institute, Russian Academy of Science, Republic of Karelia, Russia, in August 2012. All snails were brought to the laboratory and checked for the release of cercariae by placing them individually in a small amount of filtered seawater and exposing them to sunlight for 30 minutes. We used specimens of cercariae of *C. lingua *and *H. elongata *from 1 to 6 hours after emission. 

For the electrophysiological recordings, the individual cercaria was placed in a bath filled with sea water, which was thickened with microcrystalline nitrocellulose (Sigma) to restrain them in the surface level. An indifferent electrode (Ag/AgCl) established contact with the bath through saline (NaCl 170 mM, KCl 10 mM, CaCl_2_ 2 mM, MgCl_2_ 2 mM, HEPES/NaOH 10 mM, and pH 7.4). An Ag/AgCl-coupled glass microelectrode pulled by a Flaming/Brown micropipette puller (Sutter Instruments Co.) from 1B100F glass capillaries (WPI) was used as recording electrode. The microelectrode was filled with saline (an initial tip resistance of 20–40 MOhm) immediately before use and was inserted into different locations in the cercarial body and tail under transmitted light compound microscope at a magnification of ×100. The electrical signals obtained from the preparation were preamplified tenfold by a custom-built 10 GOhm input resistance headstage, amplified by an ISO-DAM amplifier (WPI, K-amp = 1000), high-pass filtered at 300 Hz, and digitized with ADC MD88 (Molodtsov V.O., 12 bit, 10 V input range, 20 kHz rate). Registration, storing, viewing, and primary data processing were performed using custom-made software [4].

To localize the cell nuclei, whole mounts of cercariae were stained with 4′-6-diamidino-2-phenylindole DAPI (1 : 1000) (Molecular Probes) for 5 minutes. The slides were examined with a Leica TCS SPE confocal scanning laser microscope with the appropriate wavelength-filter configuration settings.

## 3. Results

**Figure 1:**
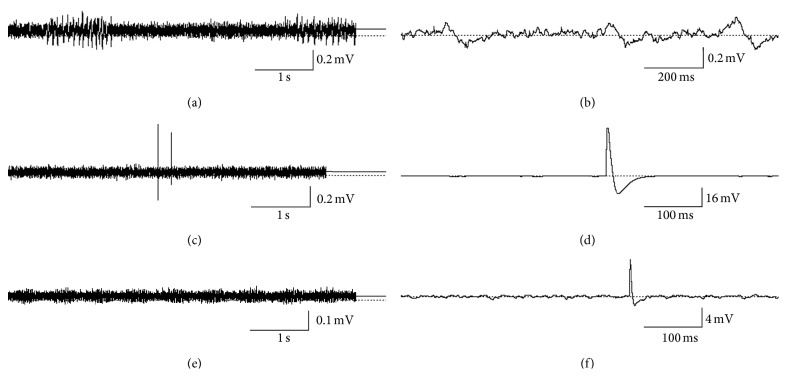
Spontaneous electrical activity in the tail of *Cryptocotyle lingua *and *Himasthla elongata *cercariae. ((a) and (b)) Slow local field potentials (sLFPS) in the middle part of the tail of *C. lingua *cercariae. ((c) and (d)) Fast action potentials (spikes) at the base of the tail of *C. lingua *cercariae. (e) sLFPs in the middle part of the tail of *H. elongata *cercariae. (f) Spike at the base of the tail of *H. elongata *cercariae.

**Figure 2:**
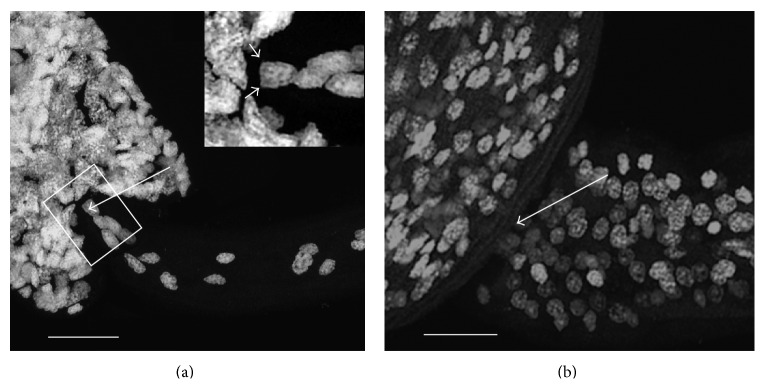
Nuclei in the tails of *Cryptocotyle lingua *and *Himasthla elongata *cercariae. Arrow—localization of electrode, scale bar = 20 *μ*m. (a) Nuclei in *C. lingua *cercaria, inset arrows—candidate spiking neurons. (b) Nuclei in *H. elongata *cercaria.

Spontaneous electrical activity was recorded in both species of cercariae. Two types of activity were found: (a, b) slow local field potentials (sLFPs) of low amplitude 0.1–0.3 mV and (c, d, f) fast high amplitude action potentials (spikes) (APs) up to about 40 mV. sLFPs were registered in the bodies and tails of both cercaria species (Figures 1(a) and 1(e)). In the tail of *C. lingua*, the sLFPs include periodical “bursts” (Figure 1(a)). In the tail of *H. elongata*, monotonous oscillations in the sLFPs were found (Figure 1(e)). Fast APs (Figures 1(c), 1(d), and 1(f)) were found in a very restricted area at the base of the tail of both cercariae (Figures 2(a) and 2(b)). In *C. lingua*, some recordings revealed two spikes of different amplitudes, suggesting that at least two neurons were discharging (Figure 1(c)). No spike trains were recorded.

In 50 cercariae of *C. lingua *studied, sLFPs were recorded in all and APs in 12 cercariae. In 49 cercariae of *H. elongata *studied, sLFPs were recorded in all, but APs were registered only in 2 cercariae.

The total number of cells in the tail of *C. lingua *is about 30 and in the tail of *H. elongata *about 330. In *C. lingua, *the area of recording included only two cells (Figure 2(a)). In *H. elongata, *the area of recording included several dozens of unidentified cells (Figure 2(b)).

## 4. Discussion

In *C. lingua*, which has an intermittent swimming behaviour, the pattern of sLFPs with the regular bursts corresponds to the electrophysiological results from *P. macrostoma *which has a similar swimming type [5, 6]. In *H. elongata*, which swims continuously, the shape of the sLFPs differs from those in *C. lingua *and *P. macrostoma *in lacking bursts. However, the mechanical method of larvae fixation does not exclude invisible movements of the muscle cells, which could be registered as sLFPs.

For the first time, APs have been detected in cercariae. The nervous system in the tail of *C. lingua *(tail size 270–420 × 33–40 *μ*m) contains a small number of paired symmetrical neurons [3]. In the region of spike recording, several pairs of candidate cells were observed with one pair of nuclei lying very close to the electrode (Figure 2(a)). It is thus likely that the recording microelectrode comes close to the spiking nerve cells or their processes. In *H. elongata *(tail size 525–560 × 45–50 *μ*m), the tenfold larger number of cells in the tail makes it difficult to approach the spiking neurons or their processes (Figure 2(b)). This might explain the few successes and the smaller amplitude of the APs recorded. In order to identify the cells and details of their interaction, further research including electron microscopy is required.

APs are a widespread signaling phenomenon in living organisms. In animals, the fast AP is usually Na^+^ based and thought to have evolved alongside the early neuromuscular systems [7]. Fast APs have been detected in free-living flatworms [8]. According to Keenan and Koopowitz [9] and Buckingham and Spencer [10], turbellarians possess a spike generation machinery similar to that in higher metazoans.

The initiation and propagation of APs place high demands on the energetic resources of the neuronal tissue [11, 12]. Although the nervous systems of both *C. lingua *and *H. elongata *contain a number of symmetrically distributed neurons, APs registered probably represent a single pair of neurons. An efficient use of spiking neurons can be recognized as an energy-saving evolutionary strategy in cercariae.

The swimming pattern of *C. lingua *cercaria consists of short periods of active tail propelling spaced with inertial motion. The two spikes (Figure 1(c)) detected in symmetrical neurons with about 200 ms delay are in a time scale of the single active phase of swimming. It is tempting to suggest that a single AP initiates the whole burst of tail propelling.

## 5. Conclusions

In the species studied, two types of electric activity are found. For the first time, fast spontaneous APs are registered in the nervous system of cercaria. The shape of the sLFPs is different in the species studied and correlates with the type of swimming, although we cannot totally exclude mechanical origin of sLFPs. The presence of few APs included in the pattern of motility in cercaria could suggest a key role for the spiking neurons in initiating the motility pattern in the cercaria and needs further research. The limited number of neurons and muscle cells in the tail of *C. lingua *cercaria makes this species a promising model for investigating the basic mechanisms of parasite motility.
